# Tracing the climate signal: mitigation of anthropogenic methane emissions can outweigh a large Arctic natural emission increase

**DOI:** 10.1038/s41598-018-37719-9

**Published:** 2019-02-04

**Authors:** Torben Røjle Christensen, Vivek K. Arora, Michael Gauss, Lena Höglund-Isaksson, Frans-Jan W. Parmentier

**Affiliations:** 1 Arctic Research Centre, Department of Bioscience, Aarhus University, Aarhus, Denmark; 2 Department of Physical Geography and Ecosystem Science, Lund University, Lund, Sweden; 3 Climate Research Division, Environment and Climate Change Canada, British Columbia, Canada; 4 Norwegian Meteorological Institute, Oslo, Norway; 5 Air quality and greenhouse gases program, International Institute for Applied Systems Analysis, Laxenburg, Austria; 6 Department of Geosciences, University of Oslo, Oslo, Norway

## Abstract

Natural methane emissions are noticeably influenced by warming of cold arctic ecosystems and permafrost. An evaluation specifically of Arctic natural methane emissions in relation to our ability to mitigate anthropogenic methane emissions is needed. Here we use empirical scenarios of increases in natural emissions together with maximum technically feasible reductions in anthropogenic emissions to evaluate their potential influence on future atmospheric methane concentrations and associated radiative forcing (RF). The largest amplification of natural emissions yields up to 42% higher atmospheric methane concentrations by the year 2100 compared with no change in natural emissions. The most likely scenarios are lower than this, while anthropogenic emission reductions may have a much greater yielding effect, with the potential of halving atmospheric methane concentrations by 2100 compared to when anthropogenic emissions continue to increase as in a business-as-usual case. In a broader perspective, it is shown that man-made emissions can be reduced sufficiently to limit methane-caused climate warming by 2100 even in the case of an uncontrolled natural Arctic methane emission feedback, but this requires a committed, global effort towards maximum feasible reductions.

## Introduction

Future concentrations of greenhouse gases (GHGs) in the atmosphere will determine the degree of warming the Earth will experience. Atmospheric methane, a powerful GHG, is controlled primarily by its anthropogenic and natural emissions and its destruction in the atmosphere. Methane is released into the atmosphere through a number of natural sources including wetlands, rivers and lakes, permafrost, wild animals, wildfires, termites, geological sources, and marine sources^1^. While significant uncertainty exists in the estimates of emissions from each of these sources, the relative order of magnitude of total global natural emissions has remained robust. This is despite discrepancies between top-down (atmospheric) and bottom-up measurement-based estimates – derived by adding estimates of individual natural methane-generating processes^2^. The rapid warming of the Arctic makes it plausible that natural emissions will increase in this area^2,3^ but the question remains whether these increases will be so large that they cannot be offset by reductions in anthropogenic emissions. To estimate the impact of future potential increases in natural methane emissions in the context of maximum technically feasible reductions in anthropogenic emissions, a modeling exercise was undertaken for this study, using an estimate of 202 Tg (±28 Tg) CH_4_/year^1^ for current global natural methane emissions. While on the lower side, this estimate lies within the range of estimates of total natural emissions (194–296 Tg CH_4_/yr) constrained by top-down approaches^1^ that take into account changes in atmospheric methane burden and methane’s lifetime in the atmosphere.

As for the Arctic itself, different assessments have recently summarized the current understanding concerning the processes that generate, consume, store and release methane in high latitude terrestrial and marine systems^2,4^, based on flux measurements, process studies, model simulations, and estimates of carbon stores. These show that both the terrestrial and marine Arctic regions are sources of methane^5^, although their current estimated magnitudes and future projections vary strongly.

For Arctic terrestrial systems, it is clear that the tundra region, inclusive of wetland areas, represents the major natural source of methane. Current estimates of natural methane emissions from Arctic tundra fall in the range of 11–39 Tg CH_4_ per year^5^. Our ability to constrain this estimate is limited by key uncertainties, primarily the limited measurements in time (few long-term records, and very limited measurements during the winter season) and in spatial coverage (very few measurement sites and most are located at “practical” rather than representative locations). Other limitations relate to gaps in understanding the biological and physical processes that control a release of methane from these ecosystems to the atmosphere. In addition to the terrestrial wetland emissions, the freshwaters of the Arctic are estimated to contribute as much as 13–16 Tg CH_4_/year^6^, yielding total terrestrial emissions in the range of 24–55 Tg CH_4_ per year i.e. about 10–25% of global natural emissions. Table 1 provides a summary of estimates for current Arctic natural methane emission sources.

**Table 1:** Natural arctic and global total current best emission estimates (CBEE)^5,16^.

Tg CH_4_/yr		Range	Best estimate
Arctic Terrestrial	Tundra	11 to 39	25
	Lakes	13 to 16	15
Arctic Marine	Sub-sea permafrost	1 to 17	5
	Sea ice leads	Not quantified	
Total Arctic		25 to 72	Ca 45
Total global natural emissions			202

Future methane releases from Arctic tundra depend on how changes in Arctic temperatures and precipitation affect these controlling processes (leading to a change in methane production or consumption), physical changes (permafrost thaw, thermokarst) as well as the extent/magnitude of available carbon pools in Arctic soils. It is estimated that 1400–1850 Pg of carbon is frozen in Arctic soils^7–9^. Since there is significant uncertainty in these estimates^10^, the potential release of this permafrost carbon to the atmosphere is also uncertain. The quantification of this uncertainty is further exacerbated due to the limited (or non-existent) representation of carbon and other biogeochemical cycle processes unique to the Arctic region and methane in Earth system models^11^. Nevertheless, it is well recognized that significant stocks of frozen carbon exist in permafrost with the potential to thaw and decay, thus releasing methane and/or carbon dioxide as the Arctic continues to warm^12^.

For Arctic marine systems, gas hydrates represent the largest potential source of marine methane release to the atmosphere due to the large amounts of methane contained within these deposits^11^. Even so, many other source types (e.g. geological) contribute to a potential release of methane from the ocean. Current emissions from the Arctic Ocean into the atmosphere are estimated to range from 1 to as high as 17 Tg CH_4_/year^13–15^. These emissions emanate primarily from shallow waters of the Arctic Ocean, since methane released from the deeper seabed is subject to significant oxidation during its ascent through the water column^1^. Future methane release from the ocean depends on the methane stocks in marine reservoirs but also on the impact of changing temperatures and sea ice coverage on the controlling biological and physical processes^2^.

A recent estimate put the quantity of carbon frozen in gas hydrates at 116 Pg^11^. This is the basis for estimating a potential increase by 1.9 Tg per year in the release of methane into the Arctic Ocean over the next 100 years^1^, which translates into an even smaller release to the atmosphere due to oxidative loss while methane rises through the water column. Overall, this is a relatively small additional contribution. However, this conclusion is tempered by significant uncertainty in the magnitude of marine hydrate carbon that is potentially vulnerable, and how much methane may pass the water column to the atmosphere, while observations that can act as a benchmark are severely lacking.

**Figure 1:**
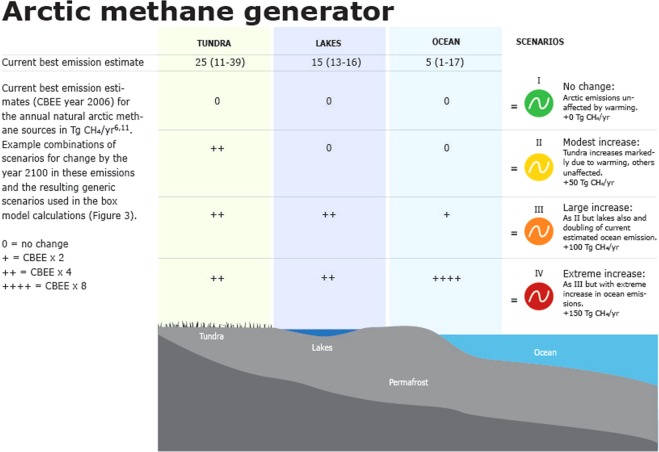
Arctic methane generator. Generic scenarios of change in natural arctic methane emissions. Scenarios I–IV are used in the box-model calculations of atmospheric concentration change pathways.

Regardless of the limitations and uncertainties in characterizing Arctic natural (terrestrial and marine) methane emissions, their impact on global and Arctic climate has the potential to be significant and needs to be quantified. The future emission values presented in Fig. 1 represent four scenarios judged to characterize the range of possible future outcomes for changes in Arctic natural methane emissions (and their different combinations). The four scenarios used to estimate the climate response to changes in natural methane emissions are described as no change (+0 Tg CH_4_/year), a low (+50 Tg CH_4_/year), a high (+100 Tg CH_4_/year), and an extreme (+150 Tg CH_4_/year) increase by 2100 above the 202 Tg CH_4_/year global natural emissions baseline^16^. We use a one box model of atmospheric methane and radiative forcing calculations to calculate impacts of future changes in methane emissions, both from anthropogenic and from natural sources, on concentrations of atmospheric methane and global climate forcing (Fig. 1, Table 2). This one box model describes the change in burden of atmospheric methane as a balance of natural and anthropogenic emissions, and the atmospheric and surface sinks (see Methods). It also models methane’s lifetime in the atmosphere as a function of its atmospheric concentration.

**Table 2:** Global radiative forcing (with respect to pre-industrial) and atmospheric concentrations of greenhouse gases with the baseline and scenarios for methane change described in this paper.

				CLE				MFR			
Year		1750	2011	2100							
Scenario				I	II	III	IV	I	II	III	IV
CH_4_	RF W/m^2^	—	0.61	1.00	1.07	1.15	1.21	0.42	0.52	0.61	0.70
	ppb	722	1803	2842	3060	3284	3511	1423	1616	1816	2021
CO_2_	RF W/m^2^	—	1.83	4.87	4.87	4.87	4.87	2.24	2.24	2.24	2.24
	ppm	278	391	670	670	670	670	421	421	421	421
Changed CH_4_ forcing with respect to CLE/scenario I	%				7	14	21	−58	−48	−39	−30

All RF values (also the ones for 2011) were calculated with the equations of Etminan *et al*.^29^.

**Figure 2:**
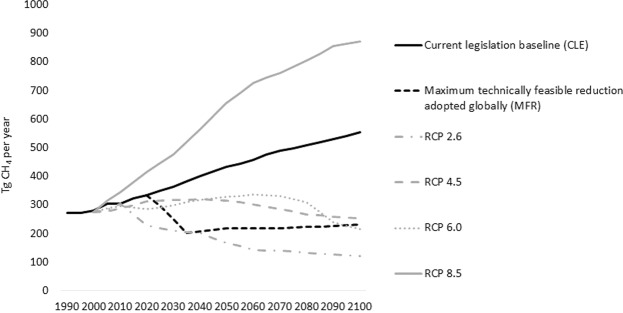
Global anthropogenic methane emissions 1990–2100 in GAINS and in the Representative Concentration Pathways (RCPs)^20,37^.

The anthropogenic methane emissions used here are updated versions of global GAINS model scenarios^17–19^ describing a current legislation (CLE) and a maximum technically feasible reduction (MFR) scenario^2^. The CLE scenario assumes no further abatement of emissions than prescribed in already adopted legislation, while the MFR scenario assumes maximum feasible implementation of existing abatement technology without considering effects of future technological development. Both scenarios fall within the ranges of the Representative Concentration Pathways^20^ (RCPs) of IPCC’s fifth assessment report^21^. For the period from 2005 to 2050 for which the GAINS model is defined, the updated CLE and MFR scenarios use macroeconomic and energy system drivers consistent with the IEA World Energy Outlook New Policy Scenario 2017^22^. For the period from 2050 to 2100, growth in activity levels for the CLE and MFR scenarios, respectively, are consistent with the Shared Socioeconomic Pathways scenarios SSP3 6.0 and SSP3 2.6^23^. For further details, see the Methods section. Figure 2 shows global anthropogenic methane emissions (excluding emissions from forest and grassland fires) over the 1990–2100 period in the CLE and MFR scenarios and in comparison with emissions for the RCP scenarios.

Within the Arctic Monitoring and Assessment Programme (AMAP, amap.no), which is one of the permanent scientific Working Groups under the Arctic Council, a multi-model study was conducted to isolate the effects of maximum feasible technical reductions in anthropogenic methane emissions on Arctic climate^24^. Three state-of-the-art Earth System Models were used, namely the Canadian Earth System Model CanESM2^25^, the Norwegian Earth System Model^26^ (NorESM), and the Community Earth System Model CESM1-CAM5 developed at NCAR, USA^27^, based on CLE and MFR emission scenarios from the GAINS model extending to 2050. The model results indicated that global implementation of maximum technically feasible reductions in anthropogenic methane is able to reduce the future average global climate warming by around 0.1–0.2 °C for the 2036–2050 period (including corresponding contributions from ozone and stratospheric water vapor), relative to a case with continuously increasing emissions according to the CLE scenario^2^. These results from Gauss *et al*.^24^ also showed that a reduction in anthropogenic methane emissions can provide a significant contribution to achieve the target of the Paris agreement, which aims to keep global warming toward the end of this century well below 2.0 °C, preferably 1.5 °C, compared to the pre-industrial level. For the Arctic region, these Earth system model results showed even larger magnitudes of reduced warming related to maximum feasible reduction in anthropogenic methane emissions (due to Arctic amplification), but also larger variability^28^. The results reported in that earlier study were based on atmospheric methane concentration changes obtained from the same one-box model of atmospheric methane that is used here. However, the Earth system model calculations in Gauss *et al*.^24^ did not consider changes in natural methane emissions and the results were analyzed only up to year 2050. Here, we use the one box model of atmospheric methane, extended to 2100, and based on the updated anthropogenic emissions for the MFR and CLE scenarios.

Arctic warming driven by all climate forcers, primarily carbon dioxide, can potentially increase natural emissions from terrestrial and marine ecosystems. In contrast, the maximum technically feasible (MFR) emission reduction from the eight Arctic Council member states alone reduces anthropogenic emissions by 142 Tg CH_4_/year by 2100 in the MFR scenario (Fig. 2), which is considerably higher than the magnitude of the potential increase by 50 Tg CH_4_/year in natural emissions due to climate warming in the low scenario but comparable in size to the increase by 150 Tg CH_4_/yr in our extreme natural emission change scenario IV.

**Figure 3:**
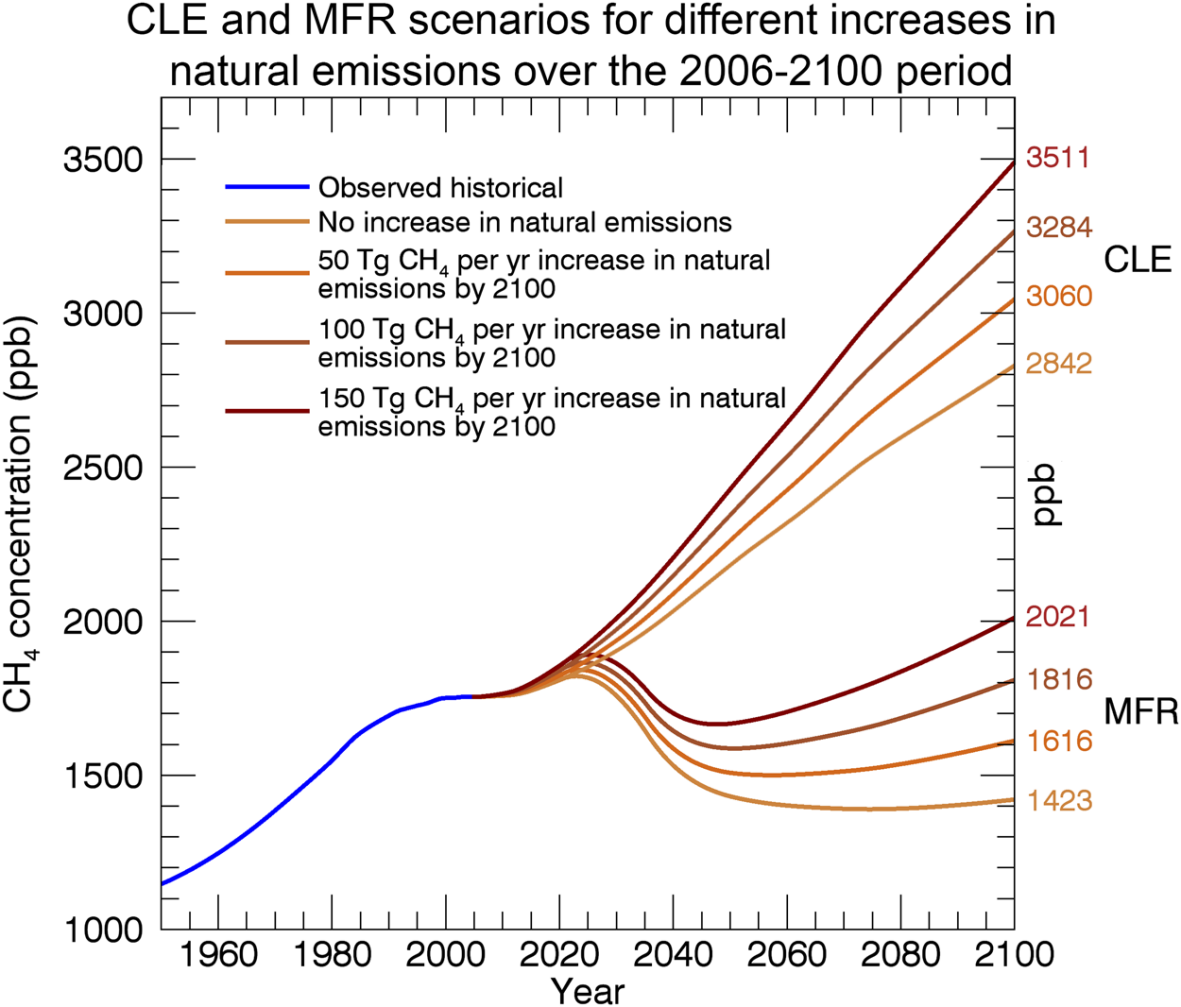
Box model results. Current legislation emission (CLE) and maximum feasible reduction (MFR) scenarios for anthropogenic impact on atmospheric methane concentrations towards 2100. The natural emission scenarios are from Fig. 1(I–IV). Each additional 50 Tg CH_4_/yr increase in natural emissions over the 2006–2100 period increase CH_4_ concentration in 2100 by about 200 ppb. But CH_4_ concentration in 2100 is reduced by about 1400–1500 ppb in response to going from CLE to MFR.

Figure 3 shows the resulting changes in atmospheric methane concentration obtained from the one box model for the MFR and CLE scenarios together with the corresponding four scenarios for changes in natural methane emissions. As Earth system models progressively include methane-related biogeochemical processes it will be possible to gain a better understanding of the effect of climate warming on methane-related feedbacks and quantify the effect of mitigation of anthropogenic methane emissions in a consistent framework. Here we use the changes in atmospheric methane concentration from Fig. 3 to calculate global-mean radiative forcing (RF) for the year 2100 for the eight different combinations of changes in anthropogenic and natural emissions, using formulae from Etminan *et al*.^29^. The results for RF, along with the concentrations used, are listed in Table 2. All CH_4_ RFs in the MFR case (0.42 to 0.70 W/m^2^) are smaller than the lowest CH_4_ RF in the CLE case (1.00 W/m^2^), since the maximum feasible reduction in global anthropogenic emissions overwhelms the natural emissions increase even in the extreme Scenario IV. The maximum increase in naturally-induced CH_4_ RF in the CLE case is 0.21 W/m^2^ (“CLE + natural emissions scenario IV” minus “CLE + natural emissions scenario I”), while in the MFR case it is 0.28 W/m^2^. The slightly higher value in the MFR case is due to lower CH_4_ concentrations (less saturation in the CH_4_ absorption bands) and lower N_2_O concentrations in the MFR case.

None of the naturally-induced CH_4_ RF increases are as large in magnitude as the decrease in CH_4_ RF range that is due to a reduction in anthropogenic emissions, represented by the differences between the CLE and MFR cases. The smallest decrease in CH_4_ RF attributable to a reduction in anthropogenic emissions is 0.51 W/m^2^ (‘CLE/Scenario IV’ minus ‘MFR/Scenario IV’) while the largest decrease is 0.58 W/m^2^ (‘CLE/Scenario I’ minus ‘MFR/Scenario I’). Also, it has to be said that in the MFR case (featuring reductions in GHG global mean concentrations), methane-caused warming in the Arctic will be lower compared to the CLE, which would make the extreme scenario IV relatively less likely than in the CLE case.

In conclusion, although sizeable for the impact on methane forcing alone, and approaching the scale of for example the cooling effect of sulphate aerosols in the atmosphere^30^, the feedback from natural methane emission changes in the Arctic remains minor compared to the effect of (global) anthropogenic emission cuts that could occur in a MFR-type scenario.

The scenarios used in this study cover a wide range of future emissions, up to an extreme scenario where the Arctic would emit an additional 150 Tg CH_4_/yr by 2100. Considering that this number is roughly equal to ~75% of current global natural emissions, such an increase appears unlikely. One of the few possible causes for such a large increase would be the widespread destabilization of gas hydrates, but recent model studies and observations indicate that this source is lower and more stable than previously thought^11,31^, and the geological record shows little evidence of large releases of methane from gas hydrates in the past^2,32^. However, terrestrial sources may increase strongly with continued warming, and Arctic climate feedbacks are not limited to methane alone. The combined release of methane and CO_2_ from the northern permafrost region represents a sustained source that can accelerate climate change^33^, while sea ice decline, snow cover loss and shrub expansion further amplify warming through a lowering of surface albedo^13^. The cumulative effect of climate change on the terrestrial and marine Arctic, and the potential for positive feedbacks that affect the rest of the world, remains a topic of high concern. Methane, however, is too often portrayed as solely being able to cause runaway climate change. Despite large uncertainties associated with future projections of Arctic natural methane emissions, our current best estimates of potential increases in natural emissions remain lower than anthropogenic emissions. In other words, claims of an apocalypse associated solely with Arctic natural methane emission feedbacks are misleading, since they guide attention away from the fact that the direction of atmospheric methane concentrations, and their effect on climate, largely remain the responsibility of anthropogenic GHG emissions.

A rise in natural methane emissions may make it more challenging to reach the goals set by the Paris agreement, but they should not be a cause for indifference. On the contrary, the possibility for natural climate feedbacks emphasizes the need for a committed and strong reduction in anthropogenic GHG emissions starting sooner rather than later to avoid dangerous climate change.

## Methods

### Box model calculations: One-box model of atmospheric CH_4_

A one-box model of atmospheric CH_4_ is used to obtain globally-averaged CH_4_ concentrations, [CH_4_], corresponding to global emissions for the CLE and the MFR scenarios. The model describes the change in burden of atmospheric CH_4_ (*H*) as a balance of surface emissions (*E* = *E*_*N*_ + *E*_*A*_, consisting of natural *E*_*N*_ and anthropogenic emissions *E*_*A*_) and the atmospheric and surface sinks (*S*).

1 $$\frac{dH}{dt}=E-S$$

The sink *S* is calculated as a first-order loss process from methane’s atmospheric lifetime in the atmosphere (${\tau }_{{{\rm{CH}}}_{{\rm{4}}}}$) as $S=H[1-\exp (\,-\,1/{\tau }_{{{\rm{CH}}}_{{\rm{4}}}})]$. ${\tau }_{{{\rm{CH}}}_{{\rm{4}}}}$ is calculated as

2 $$\frac{1}{{\tau }_{{{\rm{CH}}}_{{\rm{4}}}}}=\frac{1}{{\tau }_{{\rm{OH}}}}+\frac{1}{{\tau }_{{\rm{strat}}}}+\frac{1}{{\tau }_{{\rm{soil}}}}+\frac{1}{{\tau }_{{\rm{trop}}-{\rm{Cl}}}}$$

where *τ*_OH_ (present day value of 11.17 years), *τ*_strat_ (120 years), *τ*_trop−Cl_ (200 years) and *τ*_soil_ (150 years) are the lifetimes associated with the destruction of CH_4_ by tropospheric OH radicals, loss in the stratosphere, reaction with tropospheric chlorine and uptake by soils, respectively, following *Prather et al*.^16^ which yields a present day value of ${\tau }_{{{\rm{CH}}}_{{\rm{4}}}}$ as 9.1 ± 0.9 years in equation (2). For the pre-industrial period, *Prather et al*.^16^ estimate ${\tau }_{{{\rm{CH}}}_{{\rm{4}}}}$ as 9.5 ± 1.3 years assuming *τ*_OH_ to be equal to 11.76 years (based on Atmospheric Chemistry and Climate Model Intercomparison Project (ACCMIP) results^34^ and lifetimes associated with other processes to stay the same. In our study, the value of ${\tau }_{{{\rm{CH}}}_{{\rm{4}}}}$for the future period is calculated by changing *τ*_OH_ based on changes in [CH_4_] but *τ*_strat_, *τ*_trop−Cl_ and *τ*_soil_ are assumed to stay the same. For *τ*_OH_ we follow the approach used in the MAGICC IAM (http://wiki.magicc.org/index.php?title=Non-CO2_Concentrations) and by *Prather et al*.^16^ which results in a decrease in loss frequency ${f}_{{\rm{OH}}}=1/{\tau }_{{\rm{OH}}}$ by 0.32%, and hence an increase in methane’s lifetime, for every 1% increase in [CH_4_]. This is implemented as shown in equation (3)

3 $$0.32\,\mathrm{log}(\frac{[{{\rm{CH}}}_{4}](t)}{[{{\rm{CH}}}_{4}](t+1)})=log(\frac{{\tau }_{OH}(t)}{{\tau }_{OH}(t+1)})$$

which is used to determine methane life time associated with the destruction of CH_4_ by tropospheric OH radicals for the next time step, *τ*_*OH*_(*t* + 1), given atmospheric methane concentrations for the current and next time steps, [CH_4_](*t*) and [CH_4_](*t* + 1), and methane life time associated with OH for the current time step, *τ*_*OH*_(*t*). For the present day the value is 11.17 years and a 1% increase in [CH_4_] implies increases to about 11.21 years. This approach takes into account the positive feedback where [CH_4_] affects its own lifetime but the effects of changes in NO_x_ emissions or tropospheric water vapour are not taken into account.

The one-box model of atmospheric methane is first evaluated over the historical period to investigate if it reproduces the observation-based estimates of anthropogenic emissions given the historical rate of increase of atmospheric methane burden, and *Prather et al*.’s^16^ estimates of natural emissions and methane lifetime based on various processes associated with equation (2). After successful evaluation of the one-box model of atmospheric methane over the historical period, the model is then applied in a forward mode where given the emissions from the CLE and MFR scenarios, and the empirical scenarios of increase in natural emissions, it finds future concentrations of atmospheric methane. These results are shown and discussed in detail in the Supplementary Information.

The following scenarios I–IV described in the text are used to estimate increases in natural emissions:

- 0 Tg CH4/yr increase over 2006–2100 period (scenario I)
- 50 Tg CH4/yr increase over 2006–2100 period (scenario II)
- 100 Tg CH4/yr increase over 2006–2100 period (scenario III)
- 150 Tg CH4/yr increase over 2006–2100 period (scenario IV).

### Anthropogenic emission scenarios

The anthropogenic methane emissions used here are updated versions of previously published global GAINS model scenarios^17,35^ and include recent revisions to emission estimates and abatement potentials in the oil and gas sectors^18^ and the waste and wastewater sectors^36^. The scenarios of the GAINS model describe a current legislation (CLE) and a maximum technically feasible reduction (MFR) scenario, with the scope for future abatement with existing technology forming the difference in emissions between the two scenarios. For the period 2005–2050, the updated CLE and MFR scenarios use macroeconomic and energy system drivers consistent with the IEA World Energy Outlook New Policy Scenario 2017^22^. These take account of effects on activity levels from existing as well as announced policies, including effects of the National Determined Contributions (NDCs) made by countries for the Paris Agreement. For the period 2050 to 2100, the growth in activity drivers has been taken from the database on Shared Socioeconomic Pathways (SSPs)^23^. We find that the SSP3 “Regional rivalry” scenario corresponds the closest to the macroeconomic and population assumptions of IEA-WEO 2017 for the period leading up to 2050. The SSP3 is therefore chosen as starting point for an extension of activity levels to 2100. The drivers of the CLE scenario for 2050 to 2100 are consistent with the growth in activity levels of the SSP3 6.0, while the MFR scenario uses drivers consistent with the SSP3 2.6, which assumes lower consumption of fossil fuels. In addition to activity data changes, the MFR scenario assumes full implementation of maximum technically feasible reduction in methane emissions in the long-run. The CLE and MFR scenarios fall within the ranges of the Representative Concentration Pathways (RCPs) of IPCC’s fifth assessment report^20^. Methane emissions in the CLE scenario are lower than in the RCP 8.5, primarily because of more optimistic assumptions about transformations of the energy sector embedded in the IEA energy scenario used here for the period pre-2050. In contrast to the RCP scenarios, which include effects of technological development, the MFR scenario only considers abatement potentials from existing technology. This constrains the maximum abatement potential in the MFR scenario in the long-term.

### Radiative forcing

Values of radiative forcing (RF) for CH_4_ and CO_2_ were calculated with equations from Etminan *et al*.^29^ (their Table 1). Due to overlapping absorption, the RFs of CH_4_ and CO_2_ depend on their own concentrations but also on that of N_2_O. The RF equations require as input the reference (e.g. year 1750) and perturbed (e.g. year 2100) concentrations of CH_4_, CO_2_ and N_2_O. Concentrations of CO_2_ and N_2_O were taken from those RCP scenarios that are consistent with our GAINS anthropogenic emission scenarios, i.e. RCP 2.6 for MFR and RCP 6.0 for CLE^20^. CH_4_ concentrations are calculated using our one-box model of atmospheric CH_4_, which incorporates the whole range of maximum feasible reduction in methane emissions and also takes into account our estimates of natural emission changes.

It is important to note that in this study we have only calculated the direct RF of CH_4_ concentration change. We did not consider the indirect effects of ozone and stratospheric water vapor that would result from an increase in methane emissions. Myhre *et al*.^30^ accounted for the ozone and stratospheric water vapour contributions by multiplying the effect of CH_4_ by 1.50 and 1.15, respectively, but these scaling factors strictly apply to the pre-industrial to present-day period only. Calculating indirect effects for our future scenarios would require more advanced modelling tools but their consideration would not change the conclusions of the study, as they would increase the anthropogenic and natural contributions to methane RF by similar percentage amounts.

## Supplementary information

Supplementary information
