# Supplementary material for: Tracing the climate signal: mitigation of anthropogenic methane emissions can outweigh a large Arctic natural emission increase
Source: Sci Rep. 2019 Feb 4;9:1146. doi: 10.1038/s41598-018-37719-9 (PMC6362017; doi:10.1038/s41598-018-37719-9)
Supplement: Supplementary file 1 — Supplementary information [file 41598_2018_37719_MOESM1_ESM.pdf]

Tracing the climate signal: mitigation of anthropogenic methane emissions can outweigh a large Arctic natural emission increase

Torben Røjle Christensen[1,2], Vivek K. Arora[3], Michael Gauss[4], Lena Höglund-Isaksson[5] & Frans-Jan W. Parmentier[2,6]

1. Arctic Research Centre, Department of Bioscience, Aarhus University, Denmark.
2. Department of Physical Geography and Ecosystem Science, Lund University, Lund, Sweden.
3. Climate Research Division, Environment and Climate Change Canada, British Columbia, Canada.
4. Norwegian Meteorological Institute, Oslo, Norway.
5. Air quality and greenhouse gases program, International Institute for Applied Systems Analysis, Laxenburg, Austria.
6. Department of Geosciences, University of Oslo, Oslo, Norway.

Supplementary information

Evaluation of the one-box model of atmospheric methane over the historical period and its application for the future

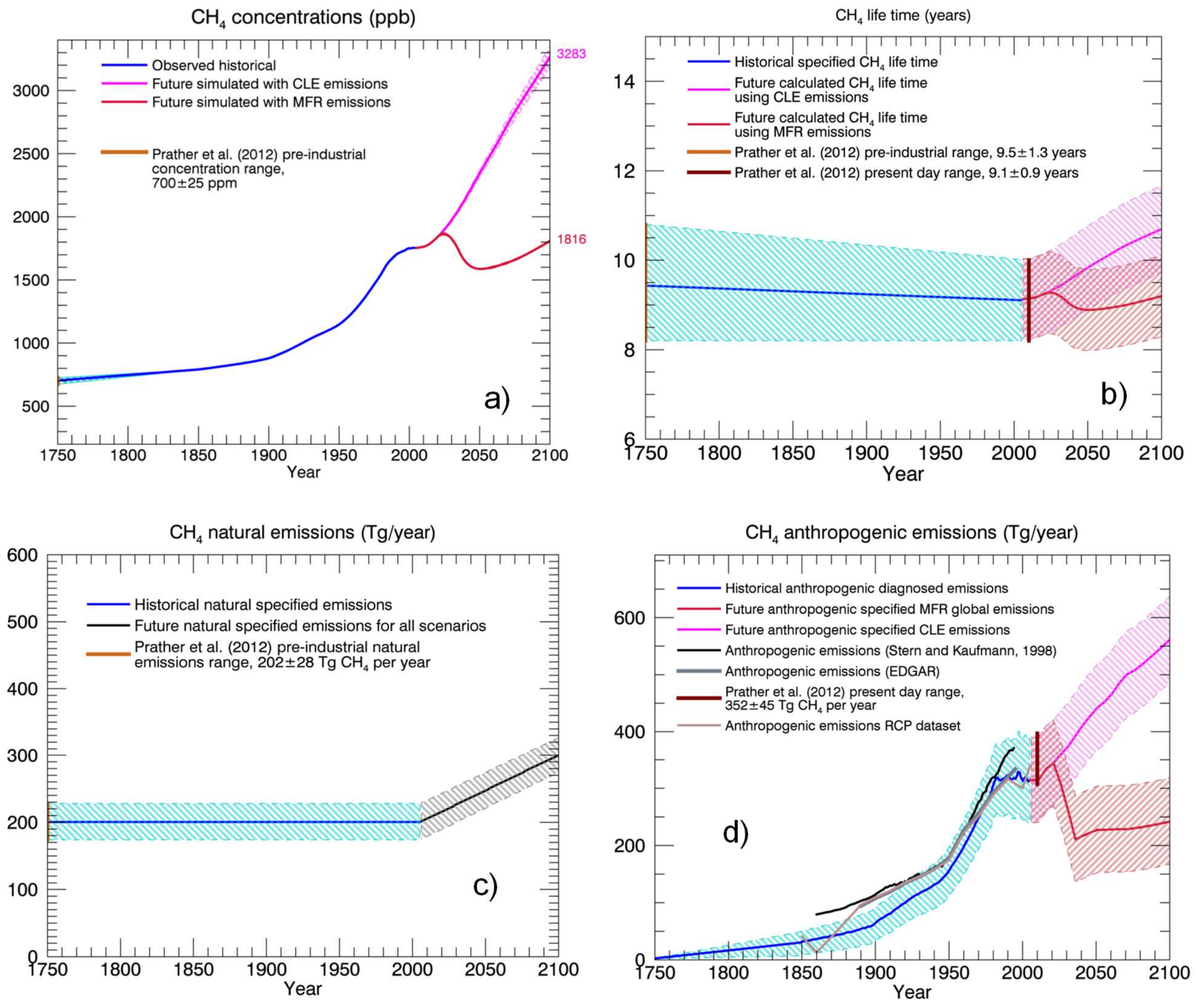

Figure S1: Evaluation over the one-box model of atmospheric methane over the historical 1850-2005 period and its application for the future 2006-2100 period as explained in the following text.

Figure S1 evaluates the one-box atmospheric CH_4 model over the historical period and shows future concentrations of atmospheric methane when the model is applied in a forward mode as discussed below. Using observation-based of atmospheric methane concentration ($[CH_4]$) (or equivalently H in equation (1) (blue line in Figure S1a), the estimates of pre-industrial and

present day τ_{CH_4} (as mentioned in the Methods section; see Figure S1b) and natural emissions E_N (202±28 Tg CH_4/yr, assumed to stay constant over the historical period), together with their uncertainties (Figure S1c), from *Prather et al.*[1] we first calculate anthropogenic CH_4 emissions E_A and their uncertainty. These are shown in Figure S1d (blue line) over the historical period together with their calculated uncertainty (blue shading). The calculated anthropogenic CH_4 emissions (in Figure S1d) for 2005 are 314±33 Tg CH_4/year (mean ± standard deviation, with a range from 239 to 399) and compare reasonably well with the observation-based estimate of 352±45 Tg CH_4/yr (mean ± standard deviation) from *Prather et al.*[1] (Figure S1c). The calculated CH_4 anthropogenic emissions over the historical period and their uncertainty range also compare well, especially after 1950, with three other inventory-based estimates (as shown in Figure S1d) from *Stern and Kaufmann*[2], EDGAR-HYDE 1.4[3] adjusted to *Olivier and Berdowki*[4] and the RCP data set (http://www.iiasa.ac.at/web-apps/tnt/RcpDb/). The uncertainty in derived anthropogenic emissions is the result of the uncertainty in τ_{CH_4} (~1.2 years, Figure S1b) and the uncertainty in natural emissions (±28 Tg CH_4/year). The 2005 anthropogenic emissions in the CLE and MFR scenario are a bit lower at 304 Tg CH_4/year (Figure 2) because they do not include methane emissions from forest and grassland fires.

Given the successful evaluation over the historical period, the one-box model of atmospheric CH_4 is then run for the future 2006-2100 period with anthropogenic emissions from the CLE and the MFR scenarios (the magenta and orange lines in Figure S1d), and the four different scenarios of increases in natural methane emissions. In Figure S1c the scenario in which natural emissions increase by 100 Tg CH_4/yr over the 2006-2100 period is illustrated, as an example. The uncertainty range in calculated anthropogenic emissions for 2005 is applied to the emissions for CLE and the MFR scenarios as well, and the emissions are increased by 10 Tg CH_4/year to be consistent with the box model's mean estimate of 314 Tg CH_4/year for 2005. The resulting atmospheric methane concentration ([CH_4]) for the CLE and MFR scenarios (magenta and dark red coloured lines, respectively) are shown in Figure S1a which shows that the difference in concentrations between the CLE and the MFR scenarios, for 100 Tg CH_4/yr increase in natural emissions, is around 1470 ppb by 2100. The lifetimes of CH_4 calculated by the box model for the

CLE and MFR scenarios, for the 2006-2100 period, are shown in Figure S1b. Simulated [CH_4] for the CLE and MFR scenarios (Figure S1a) show much smaller uncertainty than the anthropogenic CH_4 emissions (Figure S1d) and lifetimes (Figure S1b) because uncertainty in simulated future [CH_4] is tightly constrained by the assumed zero uncertainty in [CH_4] for year 2005.

The one-box model of atmospheric methane is finally run for all eight combinations of anthropogenic and natural emissions scenarios yielding the results shown in Figure 3 of the main text.

References

1 Prather, M. J., Holmes, C. D. & Hsu, J. Reactive greenhouse gas scenarios: Systematic exploration of uncertainties and the role of atmospheric chemistry. *Geophysical Research Letters* **39**, L09803-n/a, doi:10.1029/2012GL051440 (2012).

2 Stern, D. I. & Kaufmann, R. K. Estimates of global anthropogenic methane emissions 1860-1993. *Chemosphere* **33**, 159-176 (1996).

3 van Aardenne, J. A., Dentener, F. J., Olivier, J. G. J., Goldewijk, C. G. M. K. & Lelieveld, J. A 1 degrees x 1 degrees resolution data set of historical anthropogenic trace gas emissions for the period 1890-1990. *Global Biogeochemical Cycles* **15**, 909-928 (2001).

4 Berdowski, J., Guicherit, R. & Jeij, B. *The climate system*. (Balkema, 2001).
